# The Hippo signalling pathway and its implications in human health and diseases

**DOI:** 10.1038/s41392-022-01191-9

**Published:** 2022-11-08

**Authors:** Minyang Fu, Yuan Hu, Tianxia Lan, Kun-Liang Guan, Ting Luo, Min Luo

**Affiliations:** 1 Breast Disease Center, State Key Laboratory of Biotherapy and Cancer Center, National Clinical Research Center for Geriatrics, West China Hospital, Sichuan University, No. 17, South of Renmin Road, 610041 Chengdu, China; 2 Department of Pediatric Nephrology Nursing, Key Laboratory of Birth Defects and Related Diseases of Women and Children, Ministry of Education, West China Second Hospital, Sichuan University, 610041 Chengdu, China; 3 Department of Pharmacology and Moores Cancer Center, University of California, San Diego, La Jolla, CA USA

**Keywords:** Molecular biology, Molecular medicine

## Abstract

As an evolutionarily conserved signalling network, the Hippo pathway plays a crucial role in the regulation of numerous biological processes. Thus, substantial efforts have been made to understand the upstream signals that influence the activity of the Hippo pathway, as well as its physiological functions, such as cell proliferation and differentiation, organ growth, embryogenesis, and tissue regeneration/wound healing. However, dysregulation of the Hippo pathway can cause a variety of diseases, including cancer, eye diseases, cardiac diseases, pulmonary diseases, renal diseases, hepatic diseases, and immune dysfunction. Therefore, therapeutic strategies that target dysregulated Hippo components might be promising approaches for the treatment of a wide spectrum of diseases. Here, we review the key components and upstream signals of the Hippo pathway, as well as the critical physiological functions controlled by the Hippo pathway. Additionally, diseases associated with alterations in the Hippo pathway and potential therapies targeting Hippo components will be discussed.

## Introduction

**Fig. 1:**
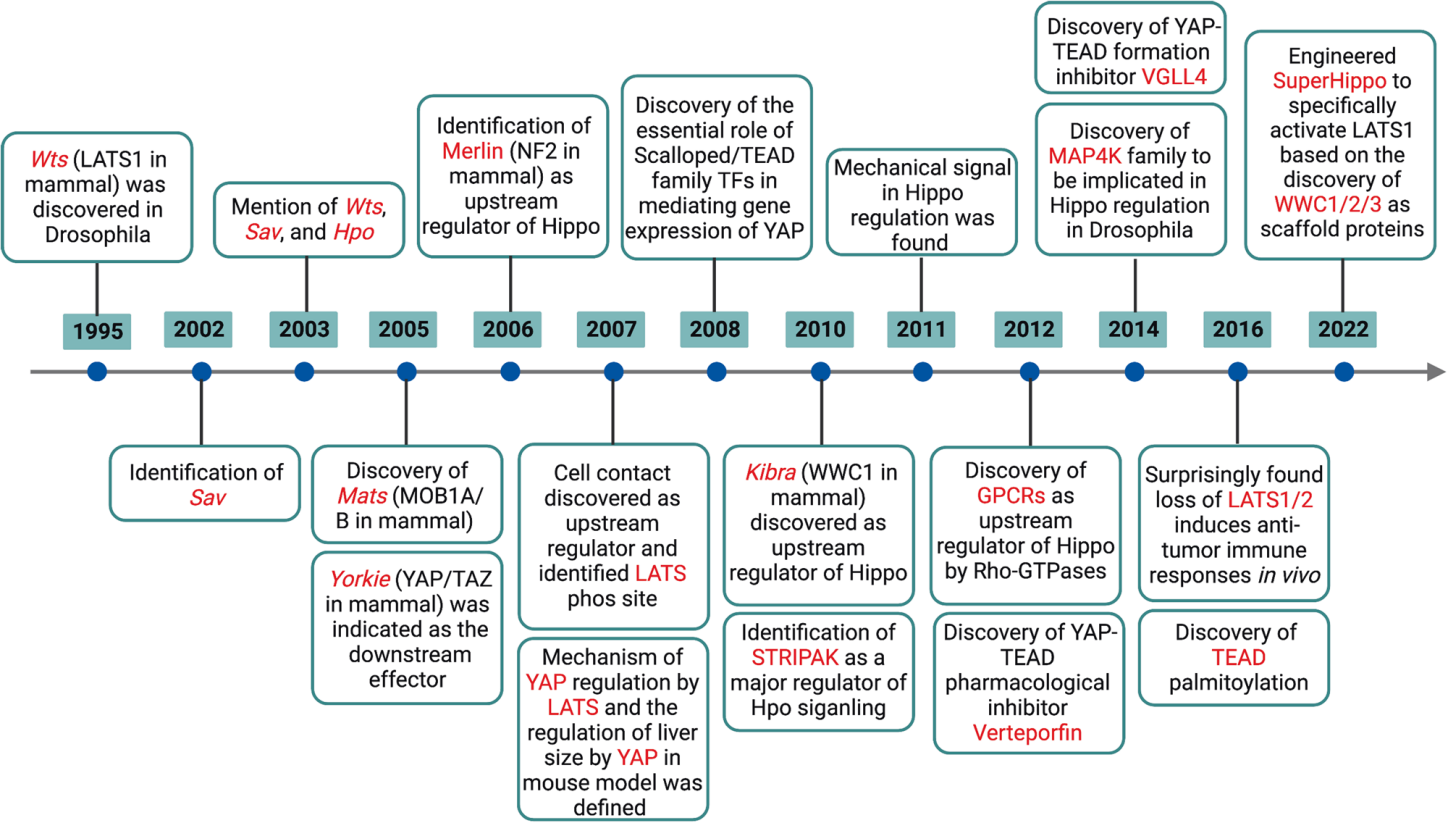
A timeline of essential discoveries and processes of the Hippo pathway. These discoveries were made initially in 1995 and then gradually to the present. The discoveries mainly focus on two aspects, including the components and processes of Hippo pathway and the function of Hippo pathway in physiological and pathological conditions

**Fig. 2:**
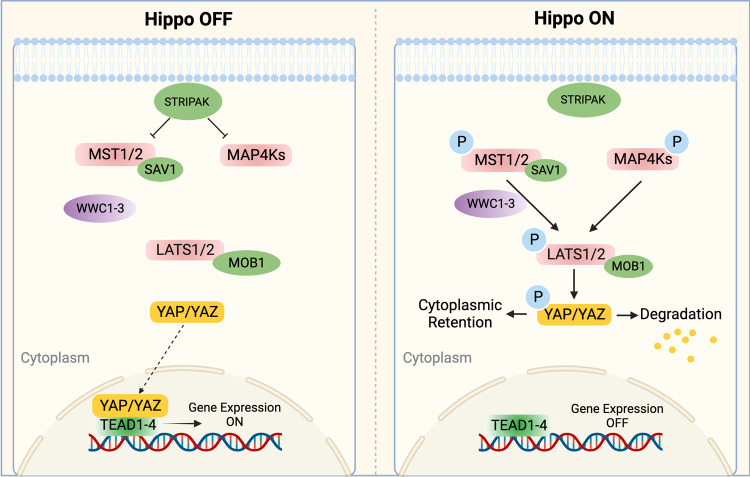
The core Hippo pathway in mammals. STRIPAK complex in the upstream regulates both MST1/2 and MAP4Ks. MAP4Ks or MST1/2 and its scaffold protein SAV1 could phosphorylate LATS1/2 and its scaffold MOB1 with the help of WWC1–3. The phosphorylated MOB1 can also directly promote the activation of LATS1/2 by inducing the conformational change of LATS1/2. The activated LATS1/2 phosphorylated and inactivated YAP/TAZ, preventing it from translocating into the nucleus and binding to transcription factors TEAD1–4

The Hippo pathway was first discovered in *Drosophila melanogaster* and has been studied for the past 20 years. A timeline of essential discoveries and processes of the Hippo pathway is shown in Fig. 1. In mammals, the Hippo pathway is composed of several key components, including mammalian STE20-like kinase 1/2 (MST1/2), protein Salvador homologue 1 (SAV1), MOBKL1A/B (MOB1A/B), large tumour suppressor kinase 1/2 (LATS1/2), Yes-associated protein 1 (YAP), WW-domain-containing transcription regulator 1 (TAZ), and the transcriptional enhanced associated domain (TEAD) family^1^ (Fig. 2). YAP/TAZ are transcriptional coactivators that bind to TEAD1–4 to regulate the expression of a wide array of genes that mediate cell proliferation, apoptosis, and stem cell self-renewal.^2^ Moreover, a variety of upstream signals, such as cell polarity, mechanical cues, cell density, soluble factors and stress signals, modulate the Hippo pathway.^3–5^

As a signalling pathway that modulates the proliferation, differentiation, and survival of cells, the Hippo pathway plays vital role in the development and homoeostasis of organs. Therefore, dysregulation of the Hippo pathway can cause a variety of diseases, including cancer,^6,7^ eye diseases,^8,9^ cardiac diseases,^10,11^ pulmonary diseases,^12,13^ renal diseases,^14,15^ hepatic diseases,^16,17^ and immune dysfunction.^18,19^ Thus, developing therapeutic approaches targeting Hippo components will expand the availability of precise therapies against cancers and other diseases. To date, a multitude of laboratory investigations has been performed to assess the therapeutic value of these strategies in vitro and in vivo. Furthermore, some of these strategies have already been evaluated in clinical trials.

In this review, key components, upstream signals of the Hippo pathway, and critical physiological functions controlled by this pathway will be discussed. In addition, studies focusing on the consequences of the dysregulated Hippo pathway and potential therapeutic strategies targeting Hippo components will be evaluated. Moreover, the outcomes of drugs that manipulate the activity of the Hippo pathway will be analysed.

### Key components of the Hippo pathway

The core of the Hippo pathway is a kinase cascade, and MST1/2, SAV1, LATS1/2, YAP, and TAZ are considered the key components.^20–22^ In general, the striatin (STRN)-interacting phosphatase and kinase (STRIPAK) complex works upstream of kinase kinase kinase kinases (MAP4Ks) and MST1/2 and inhibits the Hippo pathway.^23–26^ However, when the Hippo pathway is activated, MAP4Ks, MST1/2 and its scaffold protein SAV1 phosphorylate LATS1/2 and its scaffold MOB1A/B.^27^ Then, activated LATS1/2 phosphorylates and inhibits YAP and TAZ, preventing them from translocating into the nucleus to interact with TEAD 1–4.^28^

MST1 and MST2 are serine/threonine kinases whose activity can be enhanced by being complexed with the scaffold protein SAV1 through their C-terminal SARAH (Sav/Rassf/Hpo) domains.^29^ MST1/2 also facilitates the binding between MOB1A/B and LATS1/2.^30^ Recently, Sixian Qi et al.^31^ showed that this process was mediated by WWC proteins (WWC1/2/3), which act as scaffold proteins. Besides, except for MST1/2, MAP4K family proteins have also been reported to participate in the activation of LATS1/2 without the direct involvement of SAV.^32–35^ Thus, the activation of MST1/2 or MAP4K proteins is an initiating signal for the Hippo pathway. The combined depletion of these three types of proteins has been shown to drastically block downstream signalling.^36^

MOB1 A/B plays dual roles in Hippo activation. First, MOB1A/B functions as a scaffold that contributes to the interaction between MST1/2 and LATS1/2. Second, phosphorylated MOB1 A/B can directly promote the activation of LATS1/2 by inducing a conformational change in LATS.^37,38^ LATS1 and LATS2 are serine/threonine kinases in the AGC kinase family. Upon activation, they directly interact with downstream YAP/TAZ. It was suggested that this interaction might be mediated by WW domains on YAP/TAZ and PxY motifs on LATS1/2.^29,39,40^ Compelling evidence suggests that activated LATS1 and LATS2 phosphorylate and inactivate YAP and TAZ,^41^ the main downstream effectors of the Hippo pathway.

When the Hippo pathway is activated, the activity of YAP/TAZ is inhibited through LATS1/2-mediated phosphorylation. When the Hippo pathway is inactivated, dephosphorylated YAP/TAZ translocates into the nucleus and binds to the transcription factors TEAD1-4 to induce gene expression.^42^ TEAD1-4 could function as transcriptional repressors by recruiting vestigial-like (VGLL) family proteins, such as VGLL3^43^ and VGLL4.^44,45^ These factors competitively bind to TEAD and YAP/TAZ and cause transcriptional silencing. However, uncontrolled activation of YAP/TAZ and TEAD1-4 could lead to constitutive activation of this pathway, thereby leading to pathological consequences.^46–48^

### Upstream signals of the Hippo pathway

**Fig. 3:**
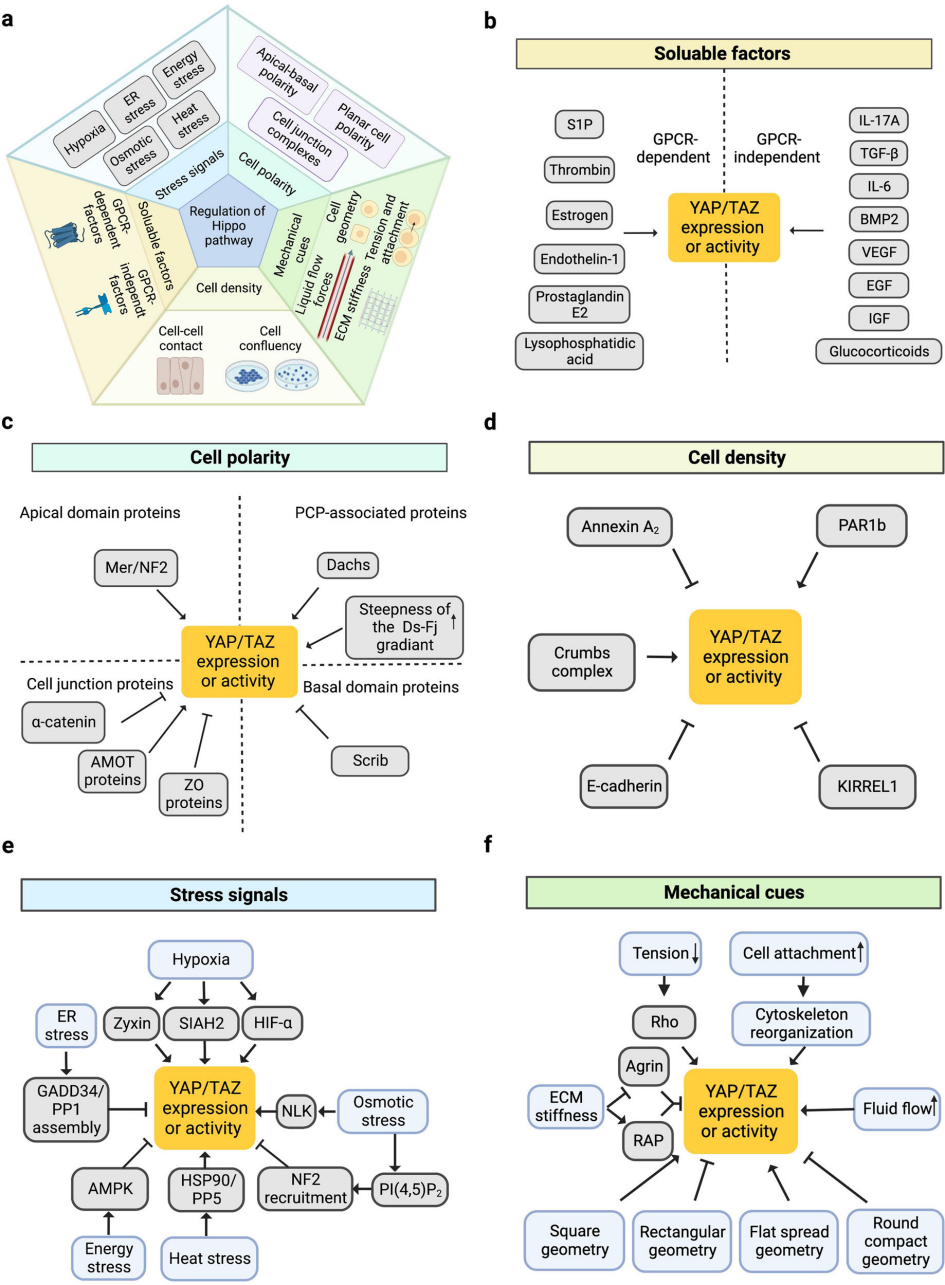
Regulation of the Hippo pathway by upstream signals. **a** Five subgroups of upstream signals including cell polarity, mechanical cues, cell density, soluble factors, and stress signals are responsible for the regulation of Hippo pathway. **b**–**f** The detailed upstream signals of Hippo pathway in every subgroup

Because of the important biological roles of the Hippo pathway, considerable efforts have been made to examine the upstream signals that regulate the Hippo kinase cascade.^5^ To date, a large number of such signals have been identified.^20,49–52^ In this review, these signals are classified into five subgroups: cell polarity, mechanical cues, cell density, soluble factors, and stress signals^53–57^ (Fig. 3).

#### Cell polarity

Cell polarity refers to distinct spatial characteristics with respect to the shape and structure of cells. It is well documented that the polarity of cells acts as a key regulator of the Hippo pathway.^29^ Generally, there are two types of cell polarity: apicobasal polarity (AP) and planar cell polarity (PCP). While AP divides the plasma membrane into the apical domain and basal domain,^58^ PCP refers to the polarization of epithelial cells along an axis perpendicular to the apical–basal axis (from proximal to distal).^59^

Notably, a wide spectrum of proteins on apical and basal domains or within the cell junction complexes have been reported to be upstream mediators of the Hippo pathway cascade.^60^ For example, in *Drosophila*, the tumour suppressor Merlin/neurofibromin-2 (Mer/NF2) localizes at the apical domain and functions as a linker for the actin cytoskeleton and plasma membrane.^61^ Mer/NF2 contributes to the recruitment of Hippo kinase and participates in the activation of the Hippo pathway.^62^ Additionally, posttranslational modifications of Mer/NF2, such as NEDD4L-mediated ubiquitination, have been shown to be required for the activation of Lats1.^63^ In mammals, it has been demonstrated that the cataract formation phenotype in NF2-deficient mice could be suppressed by the depletion of the *Yap* gene.^64^ In another study, the connection between NF2 and YAP was evaluated in the mouse myocardium.^65^ It was shown that the expression level of YAP was upregulated in the cardiomyocytes of cardiomyocyte-specific NF2-knockout mice. Moreover, NF2-deficient mice were resistant to H_2_O_2_-induced ischaemia/reperfusion (I/R) injury in the heart.^65^ In addition, *Yap* depletion could diminish the protection against I/R in cardiomyocyte-specific NF2-knockout mice, indicating that NF2 regulates the activity of YAP.^65^ Additionally, Scribble (Scrib) is a membrane protein localized on the basal domain. In *Drosophila*, the enhancement of cell migration induced by the inhibition of Scrib was shown to be mediated by Yki.^66^ Furthermore, it was shown that cancer-associated phenotypes may be induced by the interaction between Scrib and Yap, which inhibits the activity of YAP.^67^

In addition, apical and basal domains are physically separated by cell junction complexes. Many cell junction proteins have also been shown to play regulatory roles in the Hippo pathway by interacting with Hippo components.^68,69^. For example, the angiomotin (AMOT) family of proteins (AMOT, AMOTL1, and AMOTL2) are essential for tight junctions and cell polarity.^70^ It was reported that AMOT proteins function as scaffolds for LATS1/2, facilitating the phosphorylation of LATS1/2 by MST1/2. Moreover, AMOT contributes to the connection between LATS1/2 and YAP, which is required for the activation of YAP.^71^ In addition, α-Catenin is an essential component of the E-cadherin–catenin complex, and its function is of vital importance for the integrity of adherens junctions.^72^ It has been shown that α-Catenin can inhibit the nuclear localization of YAP. This inhibitory role is associated with the tumour-suppressive effects of α-Catenin.^73^ In addition, Zonula occludens (ZO) proteins (ZO-1, ZO-2, ZO-3) are scaffolding proteins that provide a structural basis for tight junctions.^74^ It has been shown that ZO-2 silencing leads to the activation of YAP and causes renal hypertrophy.^75^

Finally, PCP, another type of cell polarity, also functions as an upstream signal of the Hippo pathway. It has been demonstrated that PCP is regulated by the protocadherins Fat (Ft) and Dachsous (Ds),^76,77^ which are involved in the regulation of the Hippo pathway.^78,79^ Through the Golgi-resident kinase four jointed (FJ), Ft and Ds engage with each other heterophilically between cells. In addition, the Ft–Ds system functions as a ligand‒receptor pair for the Hippo pathway.^5^ Notably, it was shown that the regulatory effect of the Ft–Ds system on the Hippo pathway is regulated by the steepness of Ds–Fj gradients. While a shallow gradient activates the Hippo pathway, a steep gradient inhibits the activity of the Hippo pathway.^80^ Additionally, it was revealed that Dachs, an important downstream effector of Fat, plays key roles in the regulation of the Hippo pathway.^81,82^ It was reported that Dachs could influence the activity of the Hippo pathway by competing with Mats for binding to Warts.^83^

#### Mechanical cues

Mechanical cues are important signals by which cells sense their microenvironment. Through mechanotransduction systems, cells can translate mechanical cues into biochemical signals to control their behaviour. As early as 2011, Sirio Dupont et al.^84^ revealed the essential role of YAP/TAZ in the mechanotransduction system. This finding suggests a tight connection between the Hippo pathway and mechanical cues. Through the Hippo pathway, cells sense and respond to mechanical cues such as extracellular matrix (ECM) stiffness, cell geometry, liquid flow forces,^85^ tension and attachment. These mechanical cues have strong effects on the proliferation, survival, and differentiation of cells through the Hippo pathway.^84^

Changes in ECM stiffness represent an important type of mechanical cue. It was reported that the Ras-related GTPase RAP2 could be activated by low ECM stiffness, which leads to the activation of LATS1/2.^86^ Additionally, Agrin, an ECM proteoglycan that binds to lipoprotein-related receptor-4 (Lrp4) and muscle-specific kinase (MuSK), has been reported to relay matrix rigidity signals to the Hippo pathway by interrupting the functioning of Merlin and LATS1/2.^87^ Moreover, it was revealed that the aberrant expression of tenascin C leads to the repression of ECM adhesion forces and activation of the Hippo pathway, thereby facilitating new bone formation.^88^

Additionally, changes in cell geometry could affect the activity of the Hippo pathway cascade. It has been observed that YAP/TAZ tends to localize in the nucleus in murine myoblasts with a rectangular shape.^89^ When myoblasts are in an elongated rectangular shape, the ratio of cytoplasmic to nuclear YAP/YAZ is increased.^89^ This finding suggests that the geometry of cells can affect the distribution of YAP/TAZ by regulating the activity of the Hippo pathway. In addition, in NIH-3T3 mouse embryonic fibroblasts, similar geometry-mediated regulation of the Hippo pathway was identified. It was shown that YAP accumulates in the nucleus in flat spread cells, while in round compact cells, YAP localizes in the cytoplasm.^90^

Liquid flow force represents another upstream mechanical cue that regulates the Hippo pathway. It is noteworthy that a substantial proportion of human body fluids are flowing liquids such as blood and lymph.^91^ As a consequence, many cells are exposed to different levels of liquid shear stress. Compelling evidence suggests that cells respond to liquid shear forces through the Hippo pathway.^92,93^ The first study that uncovered the relationship between liquid shear forces and the regulation of the Hippo pathway revealed that increased fluid flow promoted the expression of YAP and was associated with osteogenesis and a decrease in adipogenesis in mesenchymal stem cells (MSCs). In chondrocytes, increased fluid flow leads to increased expression of YAP and results in dedifferentiation.^94^

Cytoskeleton tension and cell‒cell attachments, which are mechanical forces, are also implicated in the upstream regulation of the Hippo pathway. Several regulator proteins, such as Rho, jub and Ajuba LIMD1, take part in the transmission from tension and attachment of cells to the Hippo pathway. It was reported that Rho participates in the regulation of cell attachment-induced YAP dephosphorylation.^95^ Moreover, it was shown that the activity of Rho plays a pivotal role in the cell attachment-dependent regulation of the Hippo pathway.^96^ The protein Jub, which can negatively regulate Warts within the Hippo pathway, was shown to regulate Yki activity in response to cytoskeletal tension.^97^ In addition, the Ajuba family protein LIMD1 and the contractile protein spectrin participate in cytoskeleton tension-mediated Hippo pathway changes.^96,98^

In summary, different mechanical cues are involved in the upstream regulation of the Hippo pathway. Although it has been found that integrin,^99^ peizo^100^ and plexin^101^ can act as mechanical sensor proteins that transmit signals to the Hippo pathway, the comprehensive molecular mechanism by which cells sense mechanical signals and change cellular behaviours through the Hippo pathway is still unclear. The proteins in cell‒cell contact sites and the cytoskeleton might play critical roles in the signal transmission between mechanical cues and the Hippo pathway cascade.^102^ Notably, although the mechanisms by which mechanical cues affect the Hippo pathway cascade have yet to be fully understood at the molecular level, one of the core components of the Hippo pathway, YAP/TAZ, is a vital mediator of mechanical cues.^103^

#### Cell density

It is frequently observed that the proliferation rate of cells is negatively correlated with cell density in the monolayer culture. This phenomenon was shown to be associated with cell–cell contact.^104^ It was shown that high confluence of mammalian cells leads to the activation of LATS and the phosphorylation and inactivation of YAP. Furthermore, the overexpression of YAP could reverse the growth inhibition induced by cell density, suggesting a critical role of YAP in cell contact inhibition.^105^

The mechanisms by which the Hippo pathway is regulated by cell density have yet to be completely elucidated. Thus far, several complexes or proteins have been reported to function as sensors that transmit cell density signals to the Hippo pathway.^5,106^ It has been demonstrated that cell density can be sensed by the Crumbs complex, which includes AMOT. The Crumbs complex interacts with YAP/TAZ and facilitates its phosphorylation. This phosphorylation results in the suppression of the TGF-β-SMAD signalling pathway and leads to epithelial-to-mesenchymal transition.^68^ A recent study revealed that Kirre-like Nephrin Family Adhesion Molecule 1 (KIRREL1), a cell adhesion molecule, acts as a feedback regulator of the Hippo pathway in mammalian cells. It was shown that KIRREL1 could sense cell‒cell interactions and mediate the recruitment of SAV1 to cell‒cell contact sites. KIRREL1 knockout led to the activation of YAP.^107,108^ Recently, integrated screens revealed KIRREL as a cell surface tumour suppressor involved in the Hippo pathway that could bind directly to SAV1 to activate this pathway.^109^ In addition, it was shown that the palmitoylation of TEAD was also regulated by cell density.^110^ Reportedly, other cell density transmitters for the Hippo pathway include E-cadherin,^111^ annexin A2,^112^ and the polarity-regulating kinase PAR1b.^113^

#### Soluble factors

Soluble factors regulate the majority of biological and physiological processes. To date, numerous soluble factors have been shown to influence the activity of the Hippo pathway.^4,114^ Notably, G protein-coupled receptors (GPCRs) make up the largest family of membrane receptors for soluble factors in mammals.^115^ The Hippo pathway has been shown to be regulated by GPCR signalling.^116–119^ For example, it was shown that GPCR ligands such as thrombin or lysophosphatidic acid (LPA) could activate YAP in fibroblasts and sensitize them to TGF-β1.^120^ Additionally, sphingosine-1-phosphate (SIP) is a ligand of GPCR. SIP can induce the nuclear localization of YAP and promote the expression of YAP target genes in mouse embryonic cells and liver cells.^119^ In addition to thrombin, LPA, and S1P, a large number of soluble factors have been shown to regulate the Hippo pathway by interacting with GPCRs, such as Oestrogen,^121^ Endothelin-1,^122^ Angiotensin II,^123^ and Prostaglandin E2.^124^ Additionally, an investigation by Rui Gong et al.^125^ showed that protein kinase C (PKC) is one of the major effectors downstream of GPCRs that modulate YAP activity.

In addition, some soluble factors have been shown to affect the Hippo pathway independent of GPCRs. For example, it was reported that IL-17A could induce the recruitment of MST1 to TRAF3 interacting protein 2 (TRAF3IP2) in HaCaT and NHEK cells. Then, the MST1–LATS1 interaction is inhibited, leading to the dephosphorylation of YAP. This mechanism has been shown to facilitate cell proliferation in psoriasis.^126^ Furthermore, glucocorticoid receptor signalling has been shown to participate in the regulation of the Hippo pathway. Glucocorticoids were shown to elevate the expression of fibronectin, thus leading to cytoskeleton-dependent YAP activation in human breast cancer.^127^ Additionally, transforming growth factor-beta (TGF-β) was shown upregulate TAZ levels in mesenchymal and epithelial cells. A mechanistic study revealed that inhibiting p38 MAPK signalling suppressed TAZ upregulation in response to TGF-β.^128^ In addition, supressing myocardin-related transcription factor (MRTF) represses TAZ upregulation induced by TGF-β. These results suggest that TGF-β can regulate the Hippo pathway through p38- and MRTF-mediated signalling.^128^ Other soluble factors that influence the activity of the Hippo pathway include bone morphogenic proteins,^129^ IL-6,^130^ insulin/insulin-like growth factors,^131^ epidermal growth factors,^132^ and vascular endothelial growth factors.^133^

#### Stress signals

Cellular stress, such as hypoxia, endoplasmic reticulum (ER) stress, energy stress, osmotic stress or heat stress, can act as upstream signals of the Hippo pathway, subsequently regulating the behaviours, survival, and metabolism of cells. The mechanisms by which cells sense and transmit these signals to Hippo components have been extensively examined.^134–136^

Hypoxia is a condition in which the cell has a limited oxygen supply. In epithelial ovarian cancer cells, it was observed that 1% O_2_ or hypoxia mimics downregulated YAP phosphorylation (S127) but upregulated TAZ phosphorylation (S69), suggesting that hypoxic conditions could differentially mediate the activities of YAP and TAZ.^137^ Additionally, it has been reported that the regulation of the Hippo pathway by hypoxia is mediated by Zyxin,^138^ SIAH2 ubiquitin E3 ligase,^139^ and hypoxia-inducible factor 1 subunit alpha (HIF-1α).^140^ ER stress is induced by the accumulation of misfolded proteins in the ER when cells are exposed to an unstable or adverse environment.^141^ In human hepatocellular carcinoma cells, ER stress has been shown to inhibit the activity of YAP and enhance apoptosis by promoting the assembly of the GADD34/PP1 complex.^142^ Energy stress is characterized as a disruption of the homoeostasis of cellular energy. AMP-activated protein kinase (AMPK) functions as a sensor of energy stress. It has been demonstrated that energy stress could induce AMPK-dependent Lats activation and lead to the phosphorylation of YAP.^143,144^ This finding has been used to explain the observation that metformin, an antidiabetic drug that interacts with AMPK, exerts anticancer effects.^134^ Additionally, osmotic stress caused by sorbitol treatment can induce a dynamic balance between YAP activation and inhibition. In 2017, Hong et al.^145^ discovered that osmotic stress induced the phosphorylation of YAP at Ser128 by Nemo-like kinase (NLK), which then interfered with its binding with 14-3-3, resulting in YAP nuclear accumulation and activation. Moreover, osmotic stress inhibits YAP through phosphorylation at Ser127, and the underlying molecular mechanism was further investigated by the same team in 2020.^146^ The researchers found that osmotic stress could change the cell membrane distribution of phosphatidylinositol-4,5-bisphosphate [PI(4,5)P_2_], leading to the plasma membrane recruitment of neurofibromin 2 (NF2), also known as merlin, to induce downstream Hippo pathway activation.^146^ Moreover, heat stress is an important upstream signal of the Hippo pathway. Min Luo et al.^147^ revealed that heat stress inhibited LATS kinase by interacting with HSP90 and PPP5, thereby activating YAP/TAZ to induce the heat shock transcriptome.

### Critical physiological functions of the Hippo pathway

**Fig. 4:**
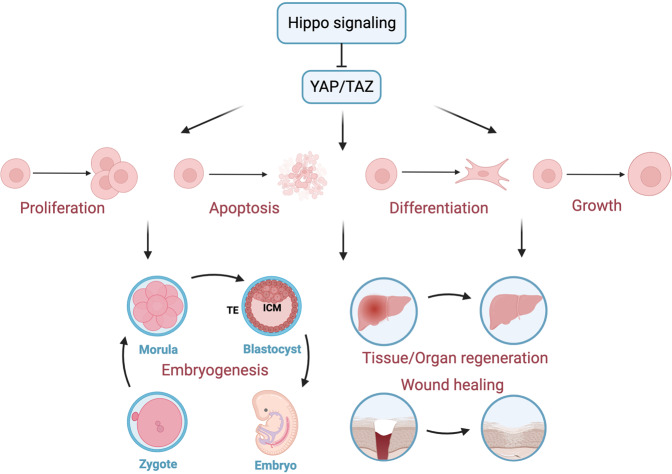
The essential physiological function of Hippo pathway. The Hippo pathway effectors YAP/TAZ can take part in the modulation of multiple cell events, including proliferation, apoptosis, differentiation and growth, thereby participating in the physiological processes of embryogenesis and development, as well as tissue/organ regeneration and wound healing

While the Hippo pathway first drew attention for its critical role in the control of organ size in *Drosophila*, over a decade of intense research has confirmed its widespread physiological roles in human health, ranging from decisions regarding cell fate determination during embryonic development to tissue/organ regeneration and wound healing (Fig. 4).

#### Cell growth, proliferation and differentiation

YAP/TAZ are key downstream effectors of the Hippo pathway that can translocate to the nucleus to induce the TEAD-mediated expression of genes related to cell growth and proliferation.^21,148^

The Hippo pathway has been shown to restrict the proliferation of cardiomyocytes,^10,149^ the molecular mechanism in which involves the Wnt^150^ or *Pi3kcb-*mediated PI3K-AKT^151^ signalling pathways. The dystrophin–glycoprotein complex (DGC) was further shown to inhibit cardiomyocyte proliferation by directly binding with Yap.^152^ Marta Diez et al.^153^ showed that 96 screened miRNAs could stimulate human iPSC-derived cardiomyocyte replication by inhibiting the Hippo pathway. In addition, in human and mouse skin, YAP-TEAD activation promotes the proliferation of keratinocytes to maintain skin homoeostasis^154,155^ or mediate IL-17A-driven psoriasis.^126^ Moreover, the Hippo pathway regulates contact-dependent cell growth and proliferation in cancer cells,^156,157^ nervous Schwann cells,^158^ epidermal stem cells^159^ and hepatic^160^ or lung epithelial cells.^161,162^

In addition to the role of the Hippo pathway in cell growth/proliferation control, this pathway has also been shown to affect specific cell differentiation in a variety of tissues and organs, including the pancreas, lung, muscle and mammary glands. For example, pancreatic-specific *Mst1/2*-knockout mice were shown to exhibit acinar cell dedifferentiation to ductal cells, which was mediated by the hyperactivation of YAP.^163^ In the developing human lung, Mst1/2 deletion and YAP activation were first shown to affect epithelial progenitor cell differentiation.^164,165^ In addition to airway epithelial progenitors, YAP is also required for the differentiation of proximal airway^166^ or airway basal stem cells^167^ in adult lungs. The role of YAP-LATS in the differentiation of type I^168^ or type II^169^ alveolar epithelial cells was further confirmed in bronchopulmonary development. However, YAP overexpression promotes myoblast differentiation^170^ and conversely represses the differentiation of satellite cells of skeletal muscle.^171^ Further studies indicated that the absence of YAP/TAZ causes the loss of the differentiated contractile phenotype in vascular smooth muscle cells (VSMCs) and osteogenic differentiation.^172^ Chen Q et al.^173^ identified that Sav1 deletion or Yap overexpression could prevent the differentiation of mammary cells. In contrast, McNeill et al.^174^ discovered that YAP/TAZ activation by Lats1/2 deletion could promote the differentiation of nephron progenitor cells into interstitial myofibroblastic cells in the kidney. In addition to the aforementioned cells, the Hippo pathway has also been reported to regulate specific cell differentiation in other tissues or organs, such as skin keratinocytes,^154^ intestinal epithelial cells,^175^ hepatocytes and biliary cells.^176,177^

Overall, these findings suggest that Hippo pathway components, especially YAP/TAZ, are involved in cell growth/proliferation and differentiation in different tissues or organs under diverse contexts.

#### Embryogenesis and development

Embryogenesis in mammals involves several essential stages, including preimplantation, gastrulation, neurulation and organogenesis. The formation of the blastocyst is one of the important events in the preimplantation stage, which consists of the outer epithelial trophectoderm (TE) and the inner cell mass (ICM). The specification of TE and ICM is a key event in early embryogenesis,^178^ and many studies have revealed the contribution of Hippo components to this process. For example, nuclear YAP was shown to be restricted in outside cells and drives TEAD4-mediated *cdx2* expression to take part in TE specification.^179^ Similarly, GATA binding protein 3 (GATA3) was shown to be regulated by TEAD4 to promote the differentiation into TE.^180^ Unlike the outside cells of the morula, WWTR1/YAP1 was repressed by LATS1/2 to permit the expression of SOX2 in the inside cells. The deletion of LATS1/2 leads to the accumulation of nuclear YAP in inner cells and causes abnormalities in the ICM and changes the cell fate towards TE-like.^181,182^ Similarly, inhibiting the upstream actor NF2 causes the mislocalization of YAP and alters the production of CDX2 inside cells.^183^ In summary, in morula, the Hippo pathway is highly activated in inside cells, whereas Hippo is in a low activation state in outside cells to maintain the normal specification of TE and ICM in the preimplantation stage.

Following the preimplantation stage, embryo development enters the gastrulation and neurulation stage on Day 26 in humans and embryonic Days 6 (E6) to 9 (E9) in mice.^178^ YAP^−/−^ embryos showed defects in the yolk sac vasculature on E8.5, which indicates that the Hippo pathway may contribute to angiogenesis in the gastrulation and neurulation stages.^184^ The relationship between angiogenesis and the Hippo pathway has already been widely discussed.^133,185^ However, the detailed underlying mechanism by which Hippo contributes to angiogenesis in this stage is unclear and should be further studied.

After the gastrulation and neurulation stages, at approximately 3–8 weeks in humans, organogenesis occurs.^178^ It has already been shown that the development of organs such as the heart, lung, and kidney is related to the Hippo pathway. In terms of cardiac development, von Gise et al.^186^ reported that YAP could contribute to cardiac development by inducing cardiomyocyte proliferation. Deletion of *Yap1* in foetal cardiomyocytes leads to lethal cardiac hypoplasia. Conversely, inactivation of the Hippo pathway, such as through *Yap1* activation or *Salv, Lats2* and *Mst1/2* knockout, causes cardiomegaly.^150,186^ For lung development, YAP is essential for the proper morphogenesis of the airway. Severe branching morphogenesis disruptions occur when the expression of YAP is blocked.^187^ Moreover, the deletion of both Mst1 and Mst2 causes severe lung abnormalities, resulting in death at birth.^164^ Furthermore, the Hippo pathway plays an important role in kidney development. It has already been reported that YAP is essential for nephrogenesis, while NF2 and LATS are needed for the morphogenesis of ureter branching.^188,189^

#### Tissue/organ regeneration and wound healing

The Hippo pathway plays pivotal roles in tissue/organ regeneration and wound healing.^190^ While tissue/organ regeneration is an important biological process that makes tissues/organs resilient to damage and disturbances, wound healing refers to the process by which the skin repairs itself after injury.^191^ Recent evidence suggests that YAP/TAZ, the key component of the Hippo pathway, is activated after damage to the skin or a variety of organs, such as the intestine, liver, heart, and lung.^192^

The role of the Hippo pathway in intestinal homoeostasis and regeneration is controversial and multifaceted.^192^ Generally, YAP/TAZ is crucial and indispensable for intestinal tissue regeneration after injury in both *Drosophila* and mice.^193^ Many studies have reported that the level of YAP protein in the intestinal epithelium is highly increased during intestinal regeneration and that its inactivation severely compromises this regenerative programme.^194,195^ In addition, the self-renewal and regeneration of the intestine are dependent on intestinal stem cells (ISCs), which are positive for leucine-rich repeat-containing GPCR5 (Lgr5)^196^ and mainly express YAP.^194^ YAP promotes intestinal regeneration by suppressing Wnt signalling in Lgr5^+^ ISCs.^197^ Conversely, an inhibitory effect of YAP on intestinal regeneration was discovered by Barry et al.^194^. The discrepancy in Wnt inhibition by YAP/TAZ may contribute to the inconsistent results. Furthermore, the inactivation of YAP/TAZ in mouse intestines resulted in no visible abnormalities, suggesting that YAP is not indispensable for normal intestinal development and homoeostasis.^195,198^

In the liver, Yap1 activation in hepatocytes contributes to liver regeneration.^199^ Additionally, the nuclear accumulation of Yap1 was increased in proliferating hepatocytes after partial hepatectomy, which facilitated the epithelial–mesenchymal transition (EMT) for liver regeneration.^200^ Additionally, blocking MST1/2 effectively enhanced liver repair and regeneration.^201^ It is commonly believed that the regenerative capacity of mammalian hearts is lost after the neonatal stage.^202^ However, recent evidence suggests that YAP activation can induce cardiac regeneration in adult mice.^10,190,203,204^ Several investigations have been conducted to examine the mechanisms by which YAP/TAZ mediate cardiac regeneration. For example, Yap activates the insulin-like growth factor (IGF) signalling pathway to augment the proliferation of cardiomyocytes.^205^ In addition, Yap activation was shown to be associated with EMT during cardiac regeneration.^206^ In the context of lung regeneration, it was reported that in alveolar stem cells, Yap is activated after pneumonectomy, which plays a critical role in alveolar regeneration.^207^ Moreover, it was observed that lung regeneration is substantially delayed in mice that lack Yap/Taz in alveolar epithelial type II cells.^208^ In addition to these organs, activation of YAP/TAZ has been shown to contribute to regeneration in many other tissues, such as the nervous system^209–211^ and bone.^212^

The roles of the Hippo pathway in wound healing have been extensively examined.^213–215^ Notably, the Hippo pathway affects wound healing through various mechanisms. For example, the activation and nuclear localization of YAP/TAZ promote proliferation in epithelial cells during wound healing.^213,216^ Additionally, TEAD inhibition increases Kruppel-like factor 4 (KLF4) levels and disrupts skin homoeostasis, thus impairing wound healing.^154^ In addition, wound-healing-related epithelial–mesenchymal transition (EMT) was shown to be regulated by YAP/TAZ in mice.^217^ Furthermore, the inhibition of YAP was shown to promote the expression of IL-33 and then lead to autophagy inhibition, which contributes to wound healing.^218^

Collectively, embryonic development and adequate and efficient tissue regeneration require highly controlled and harmonious cell proliferation, as well as cell differentiation. Considerable efforts should be devoted to understanding the complex molecular mechanisms by which YAP/TAZ mediate these critical physiological functions.

### Dysregulation of the Hippo pathway and human diseases

**Fig. 5:**
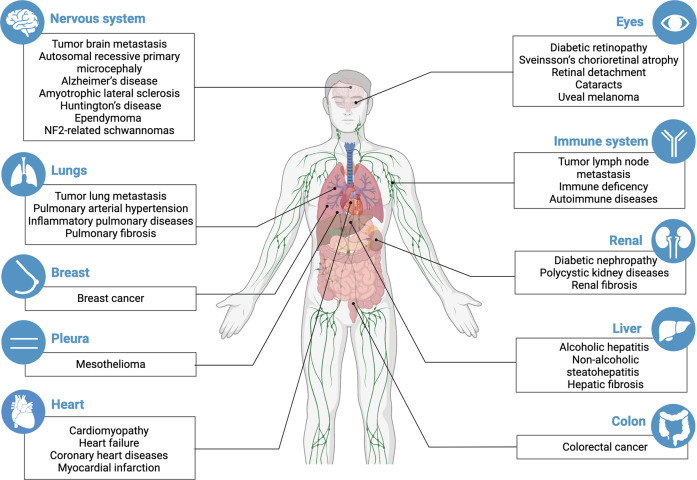
The summary of diseases caused by the dysregulation of the Hippo pathway. Hippo pathway dysregulation has been found to be present in a variety of organs or systems diseases and involved in the regulation of occurrence or progression of these diseases. The specific diseases are shown in boxes

As a signalling pathway that governs the proliferation, differentiation, and survival of cells, the Hippo pathway plays a vital role in the development and homoeostasis of organs. Therefore, dysregulation of the Hippo pathway causes a variety of diseases, including cancer, eye diseases, cardiac diseases, pulmonary diseases, renal diseases, hepatic diseases, and immune dysfunction (Fig. 5). In this section, the consequences of aberrant Hippo pathway function in human diseases will be discussed.

#### The Hippo pathway in cancer

The hypothesis that the Hippo pathway has a close connection with cancer was initiated by the discovery that the egregious overgrowth of *Drosophila melanogaster* tissues could be caused by Hippo gene mutations.^219–221^ Overwhelming evidence suggested that the Hippo pathway was one of the most frequently dysregulated pathways in human cancer; YAP/TAZ were commonly identified as oncoproteins, while MST1/2 and LATS1/2 were identified as tumour suppressors.^222–225^ Cancers such as uveal melanoma,^226^ mesothelioma,^227^ ependymoma,^228^ and NF2-related schwannomas^229^ have all been shown to be related to Hippo pathway dysregulation. Generally, the Hippo pathway can affect human cancer in three ways: tumour initiation and progression, tumour metastasis, and tumour drug resistance. The detailed underlying mechanisms will be discussed below.

##### The Hippo pathway in tumour initiation and progression

Tumour initiation and progression is a multistep process that is characterized by the transformation of normal cells to malignant tumour cells and is triggered by multiple factors. One of these factors is the dysregulation of signalling pathways related to cellular survival.^230^ Because the Hippo pathway is an essential survival-associated signalling pathway, inactivation of this pathway could increase cell proliferation and decrease apoptosis, contributing to tumour initiation and progression.^3,219^ Tumour initiation and progression require metabolic reprogramming.^231^ Some researchers have reported that the Hippo pathway participates in cancer-related metabolic reprogramming, such as glycolysis, which requires tumour cells to obtain the necessary energy and building blocks and can be promoted by active YAP.^144,232^

Cancer stem cells (CSCs), which are a subpopulation of cancer cells, play an important role in tumour initiation and progression.^233^ In a recent study, the Hippo pathway component TAZ potentiated CSCs. Depletion of TAZ significantly decreased the tumour-seeding ability.^234^ Thus, the Hippo pathway contributes to tumour initiation and progression by regulating CSCs.

However, a few recent studies have presented some opposing ideas. In certain types of tumours, the Hippo pathway may change from a tumour suppressor to a tumour promoter. Cheung et al.^235^ found that YAP plays a suppressive role in colorectal tumour growth. YAP overexpression hindered both primary and metastatic colorectal cancer. Similar tumour suppressive activity of YAP/TAZ was observed in ER^+^ breast cancer,^236^ haematological cancers^237^ and several solid cancers of neural/neuroendocrine origin.^238^ In addition, deletion of the tumour suppressor’ LATS1/2 in cancer cells inhibits tumour growth in B16, SCC7 and 4T1 immunocompetent syngeneic mouse models in vivo due to enhanced immunogenicity of the cancer cells.^41^ These findings suggest that the Hippo pathway may play a tissue type-specific role in tumorigenesis. Further evaluation of this relationship in the occurrence and development of different types of tumours is essential for the development of precise Hippo-related treatments.

##### The Hippo pathway in tumour metastasis

Tumour metastasis is known as a multistep process that is associated with a higher level of malignancy. Accumulating evidence suggests that Hippo pathway components, including LAT1/2, MST1/2, YAP and TAZ, may play important roles in influencing tumour metastasis.^235,239–244^ Furthermore, the Hippo pathway was shown to influence various types of metastases. Lung and lymph nodes are two common sites of breast cancer metastasis, while the brain is a common site of lung cancer metastasis.^245,246^ in *YAP-*deficient *PyMT* mice, which is a breast cancer model, the incidence of lung metastasis was reduced.^173^ In a breast tumour with high lymph node metastasis, the expression of *LATS1/2* decreased cancer metastasis.^241^ In addition, YAP inhibition could markedly decrease H2030-BrM3 cell brain metastasis in vivo.^247^ In most cases, the Hippo pathway may act as an inhibitor of tumour metastasis; thus, it represents a potential target for antitumor metastasis therapies.

The underlying mechanism by which the Hippo pathway affects tumour metastasis can mainly be divided into two parts. First, the Hippo pathway can regulate the migration and invasion of cells. Epithelial-to-mesenchymal transition (EMT) is a significant characteristic of cancer cells with enhanced migration.^248^ Activated YAP/TAZ can increase the expression of EMT-related transcription regulators.^20,249^ In contrast, Hippo pathway inactivation can play suppressive roles against the migration and invasion of cells. For example, YAP knockout promotes breast cancer lung metastasis.^194,250,251^ Second, the Hippo pathway could contribute to tumour metastasis by suppressing anoikis, a form of apoptosis induced by the loss of attachment between cells and the ECM. It was revealed that LIM domain only 3 (LMO3) could inhibit anoikis to promote hepatocellular carcinoma metastasis by suppressing the Hippo pathway.^252^

##### The Hippo pathway in the development of tumour drug resistance

Multiple therapies have been developed to treat cancers. In particular, chemotherapy, immunotherapy, and targeted therapy are the three main anticancer therapies. However, the efficacy of these treatments is severely impaired due to tumour drug resistance.^253–255^ Compelling evidence has shown that the Hippo pathway contributes to the development of chemotherapy resistance. The main four Hippo components, YAP, TAZ, MST1 and LATS1/2, were all shown to take part in the development of chemoresistance.^256^ For example, YAP and TAZ overexpression or YAP nuclear translocation could decrease the efficacy of cisplatin,^257^, doxorubicin,^258^ 5-fluoracil^259,260^ and Taxol,^261^ while MST1 and LATS1/2 downregulation resulted in resistance to cisplatin^262^ and 5-fluoracil.^263^

The underlying mechanism of chemotherapy resistance may be connected to the stemness of cancer cells or drug metabolism and efflux. First, the Hippo pathway could affect chemotherapy resistance by regulating CSCs. CSCs, which are tumour-initiating cells, are a subgroup of cancer cells that could contribute to chemoresistance. The Hippo pathway is an important pathway that regulates CSCs^234,264–266^. In ovarian cancer, the overexpression of miR-30b and the downregulation of *MYPT1* were shown to cause the expansion of CSCs by inactivating the Hippo pathway, ultimately resulting in platinum resistance^264^. In addition, the Hippo pathway was shown to be related to drug metabolism and efflux, which is an important factor in determining the efficacy of chemotherapy drugs. The increased efflux and metabolic conversion of a drug will decrease its efficacy considerably by reducing intracellular drug concentrations^267^. It was reported that YAP activation could sensitize pancreatic cancer cells to gemcitabine by downregulating drug efflux transporters and decreasing the conversion of gemcitabine from a less active form to an active form, resulting in increased concentrations of gemcitabine in tumour cells^268^.

In addition to chemotherapy, immunotherapy can be affected by the Hippo pathway. Over the last decade, immunotherapy has been widely examined. Immune checkpoint inhibitor therapy and chimeric antigen receptor (CAR) T-cell immunotherapy are the most striking examples^269–271^. However, the efficacy of these therapies still faces challenges associated with resistance. Myeloid-derived suppressor cells and tumour-associated macrophages are immunosuppressive cells in the tumour microenvironment that can contribute to immunotherapy resistance^272–274^. It was reported that the Hippo pathway could influence these two cell types to reduce the efficacy of immune therapy. YAP can directly induce cytokines such as CXCL5 and CCL2, which attract myeloid-derived suppressor cells and tumour-associated M2 macrophages, respectively, to confer resistance to immunotherapy^275,276^.

Finally, the most important clinical implication is the involvement of the Hippo pathway in targeted therapy resistance. There are currently several targeted cancer therapies, such as BRAF inhibitors, MEK inhibitors and epidermal growth factor receptor (EGFR) tyrosine kinase inhibitors. BRAF inhibitors can be used to treat BRAF-mutant melanoma. However, efficacy could be limited because of drug resistance.^277,278^ While exploring the mechanism of BRAF inhibitor resistance in melanoma, researchers found that the upregulation of MOB3B and activation of the Hippo pathway contribute to vemurafenib resistance.^279^ In addition, *NF2* is involved in vemurafenib resistance.^280^ Regarding MEK inhibitor resistance, the Hippo pathway may play an essential role. In vitro, the A549 and HCC44 cells that express YAP1 5SA were shown to have selumetinib resistance.^281^ The combination of YAP suppression and MEK inhibition can induce apoptosis in NSCLC, melanoma, colon cancer and thyroid cancer harbouring BRAF V600E, while the MEK inhibitor alone can not induce this effect.^282^ Clinically, the increased YAP levels may decrease the efficacy of MEK inhibitors.^282^ Ultimately, in terms of EGFR tyrosine kinase inhibitor resistance, TAZ expression is one of the intrinsic mechanisms. The overexpression of TAZ in PC9 cells reduced their sensitivity to gefitinib. TAZ knockdown sensitized gefitinib-resistant PC9/GR cells to gefitinib.^283^ Moreover, other targeted therapies, such as mTOR and CDK4/6 inhibition therapy, were shown to be related to the Hippo pathway.^284,285^

#### The Hippo pathway in eye diseases

The connection between the Hippo pathway and the development of the eye has been studied for many years. In many ocular tissues such as the cornea, lens and retina, YAP was shown to be ubiquitously distributed.^286^ Additionally, it was reported that the Hippo pathway may play an indispensable role in regulating retinogenesis, retinal neurogenesis, retinal angiogenesis, and corneal wound healing, suggesting that the Hippo pathway is involved in the regulation of ocular development.^287–290^ Therefore, dysregulation of the Hippo pathway substantially disrupts eye homoeostasis and results in different types of eye diseases.

In general, changes in the Hippo pathway in eye diseases are relatively complex. Hippo pathway dysregulation seems to be common in retinal-related diseases. In the retinas of mice with diabetic retinopathy, LATS and TAZ were increased, and p-MST and p-YAP were significantly decreased.^291^ In addition, in patients with Sveinsson’s chorioretinal atrophy, the Tyr421His mutation in *TEAD1* has been found, and this mutant Tyr421His TEAD1 has a compromised interaction with YAP.^148,292^ Moreover, MST2 but not MST1 was identified as a factor that causes retinal detachment-induced photoreceptor cell death. MST2 deficiency could prevent photoceptor cells from death after retinal detachment.^293^

In addition to retinal-related diseases, the heterozygous inactivation of *YAP1*, which decreases the expression of YAP1 protein in lens epithelia, results in cataracts with lens epithelial cell defects.^294^ Moreover, NF2 deficiency in the lens epithelium in mice could lead to a cataract phenotype.^64^

#### The Hippo pathway in cardiac diseases

As early as 2011, a series of studies uncovered the role of the Hippo pathway in regulating heart size.^150,205^ Moreover, Wei Yu et al. identified the vital role of VGLL4 in heart valve development. The deletion of VGLL4 in mice could lead to serious valve malformation.^295^ These studies resulted in further studies on the connection between cardiac regulation and the Hippo pathway. Among them, the effect of the Hippo pathway on cardiac diseases is a hot topic. Cardiac diseases that are associated with a dysregulated Hippo pathway could be divided into at least four types: cardiomyopathy; heart failure; coronary heart diseases; and myocardial infarction.^296^

Cardiomyopathy is a group of cardiac diseases that are characterized by myocardial dysfunction. Hypertrophic cardiopathy (HCM), arrhythmogenic cardiomyopathy (ARCV) and dilated cardiomyopathy (DCM) are three common cardiomyopathies.^297^ All are associated with Hippo pathway dysregulation. Samples from patients with HCM were investigated, and YAP was shown to be increased at both the protein and mRNA levels. Moreover, the inhibitory phosphorylation at Ser127 of YAP was decreased, indicating the activation of YAP.^298^ However, the condition seems to be different in ARCV^299^ and DCM,^300^ in which the Hippo pathway was activated, leading to YAP inactivation.

Heart failure is a functional and structural heart disorder with complex clinical syndromes that can be affected by the Hippo pathway. p-YAP and p-LATS were increased in ischaemic or nonischaemic heart failure samples.^301^ In addition, TEAD1 was reported to take part in the dedifferentiation of cardiomyocytes to exacerbate heart failure during pressure overload.^302^ The above studies suggest that heart failure is often accompanied by abnormal Hippo pathway activation.

In addition, the progression of coronary heart diseases and myocardial infarction may be related to changes in the Hippo pathway.^296^ YAP/TAZ activity is involved in atherogenesis, which is a characteristic of coronary heart disease.^303^ Interestingly, specific deletion of YAP in fibroblasts can effectively reduce the fibrotic response and improve cardiac function after myocardial infarction.^304^

#### The Hippo pathway in pulmonary diseases

The COVID-19 outbreak has resulted in a substantial focus on the relevant mechanisms of the development of lung diseases. As a signalling pathway that could take part in pulmonary development, the Hippo pathway may contribute to pulmonary diseases when it is dysregulated.^162,305^ Generally, dysregulation of the Hippo pathway frequently occurs in the progression of three pulmonary diseases: pulmonary arterial hypertension, inflammatory pulmonary diseases, and pulmonary fibrosis.

First, Hippo components such as YAP and LATS1 are dysregulated in pulmonary arterial hypertension. The inactivation of Hippo could induce proliferation and suppress apoptosis in pulmonary arterial smooth muscle cells (PASMCs), which is one of the most important factors in vasculature remodelling in pulmonary arterial hypertension.^306,307^ Interestingly, recent research revealed that MST1/2, a Hippo component that usually plays an antiproliferative role, supports the abnormal proliferation of PASMCs in pulmonary arterial hypertension via forehead homeobox type O and BUB3.^308^

Second, the Hippo pathway could regulate the inflammatory response in the lung. In a bacterial pneumonia model, the relative levels of p-YAP and p-TAZ were decreased in alveolar epithelial type II cells (AECIIs). YAP/TAZ activation seemed to be a protective reaction. When YAP/TAZ was deleted in AECIIs, inflammation in the lung became more severe.^208^ Similarly, the deletion of YAP in lung endothelial cells could lead to inflammation in the lung.^309^ In summary, activation of the Hippo pathway may contribute to pulmonary inflammation. However, the connection between other Hippo components and pulmonary inflammation needs to be further studied.

Third, Hippo can contribute to the progression of fibrotic diseases in the lung. Pulmonary fibrosis is the result of many interstitial pulmonary diseases and is associated with activated fibroblasts and exuberant extracellular matrix deposition. YAP promotes the proliferation and migration of fibroblasts, inducing the production of collagen and inhibiting epithelial cell differentiation, thus contributing to the progression of idiopathic pulmonary fibrosis.^310,311^

#### The Hippo pathway in renal diseases

Renal diseases have been recognized as a serious public health burden in the past decade.^312^ Chronic kidney diseases have a widespread impact and have received much attention. Diabetic and polycystic kidney diseases are two types of chronic kidney diseases.^15,313^ They are all related to dysregulation of the Hippo pathway.

In diabetic nephropathy, the abnormal proliferation of glomerular mesangial cells is one of the pathological characteristics. The Hippo pathway is inactivated and contributes to abnormal proliferation.^314^ In addition, in renal enlargement, which is one of the early structural changes in diabetic nephropathy, the Hippo pathway takes part in regulating the proliferation of tubular epithelial cells.^315^

Except for diabetes, many recent studies have highlighted Hippo-YAP signalling in renal cyst formation to explain the contribution of the Hippo pathway to polycystic kidney diseases, which are inherited disorders mainly caused by *PKD1* or *PKD2* mutations. It was reported that PKD1 deficiency in mice resulted in YAP and TAZ activation in cystic tubular epithelia. Knockout of YAP and TAZ in the autosomal dominant polycystic kidney disease model significantly suppressed cystogenesis.^316,317^ In the *pkd2* morphants, the Hippo pathway was inactivated as well, resulting in YAP dephosphorylation and nuclear translocation.^318^

Furthermore, the Hippo pathway could take part in the formation of a fibrotic environment in the kidney. Renal fibrosis is a pathophysiological hallmark of patients with chronic kidney diseases. Ischaemia‒reperfusion (IR) injury is a common model used to study the acute renal injury-chronic kidney disease transition. In the IR injury model, YAP levels were increased along with the progression of renal fibrosis.^319^ In other renal injury models with renal fibrosis, such as the obstructive, aristolochic acid and diabetic nephropathy models, the expression of TAZ in the tubulointerstitium was elevated.^320^ In addition, MST1/2 deletion in renal tubule cells caused progressive renal interstitial fibrosis.^319–321^

#### The Hippo pathway in central nervous system disorders

Central nervous system disorders are traditionally classified as early-onset neurodevelopmental and late-onset neurodegenerative diseases.^322^ Hippo pathway dysregulation is closely associated with neurodegenerative diseases.^323^

In neurodegenerative diseases such as Alzheimer’s disease (AD), amyotrophic lateral sclerosis (ALS) and Huntington’s disease (HD), the main characteristic is the abnormal death of functional neural cells, which could be mediated by the Hippo pathway.^324^ In AD, the Hippo pathway seems to be activated. One of the causes of AD is the accumulation of amyloid-beta 42 peptides (Aβ42). The activated Hippo pathway could contribute to Aβ42-induced neural cell death.^325^ Additionally, in Fused in Sarcoma (FUS)-mediated ALS, activated Hippo participated in neuronal cell death by further activating c-JUN amino-terminal kinase (JNK). Cell death could be rescued by downregulating the Hippo pathway.^326^ In SOD1(G93A) mice, which are a commonly used mouse ALS model, genetic deletion of *MST1* could improve spinal cord motor neuron viability and decrease mortality.^327^ Finally, in HD mice, deficiency in TEAD/YAP-dependent transcription could lead to necrotic cell death.^328^ In addition, LATS was activated in the brains of patients with HD.^329^

#### The Hippo pathway in hepatic diseases

The Hippo pathway not only affects the physiological regeneration of the liver but also impacts the progression of liver disease. Among the liver diseases that the Hippo pathway can influence, hepatitis and liver fibrosis are two of the most well-documented examples.^17,200,330^

Alcoholic hepatitis (AH) and nonalcoholic steatohepatitis (NASH) are two kinds of hepatitis that can be regulated by the Hippo pathway. In a mouse model of AH,^331^ YAP levels were elevated in hepatocytes, while in liver samples from human patients with AH,^332^ YAP1 mRNA was increased, and the active form of MST1 was decreased, indicating low Hippo pathway activity in AH livers. Similarly, the levels of TAZ and YAP were elevated in mice and human patients with NASH.^333^ One of the underlying mechanisms by which YAP/TAZ is increased in NASH may be related to the suppression of TAZ degradation mediated by β-TrCP-mediated ubiquitination and degradation.^334^

Hepatic fibrosis results from chronic liver damage, which can be caused by NASH, alcohol abuse or hepatitis virus infection.^335^ Carbon tetrachloride (CCl_4_) is a hepatotoxin that is widely used to establish hepatic fibrosis animal models.^336^ In hepatic fibrosis caused by CCl_4_ injection, YAP was increased in the nucleus and cytoplasm in hepatocytes and biliary cells from fibrotic livers. After YAP deletion, the expression of *Col1a1* was reduced, suggesting the suppression of fibrogenesis.^337^ Similarly, Jie et al.^338^ found that a dopamine receptor D2 antagonist, which could block YAP in macrophages, had the potential to attenuate CCl_4_-induced liver fibrosis. However, in the IR injury model, YAP plays a protective role against IR stress and decreases IR-induced liver fibrosis.^339^ Therefore, YAP has a complex role in liver fibrosis that depends on cell type and context.

In terms of the underlying mechanism, the Hippo pathway could contribute to hepatitis and hepatic fibrosis by regulating the activation of hepatic stellate cells. Hepatic stellate cells are important hepatic cells that can secrete various inflammatory molecules and extracellular matrix components to aid the progression of hepatic inflammation and fibrosis.^340,341^ The effect of YAP/TAZ on the activation of hepatic stellate cells has been verified in multiple studies, supporting the importance of the Hippo pathway in the progression of hepatic inflammation and hepatic fibrosis.^333,337,342^

#### The Hippo pathway in immune dysfunction

The immune system consists of two parts: the innate immune system and the adaptive immune system. In the past few years, it was observed that dysregulation of the Hippo pathway can influence both innate and adaptive immunity.^18^

Innate immunity is the first line of defence that protects the body from infection in a nonspecific way, and the Type I interferon (IFN) response, which is known as IFNα and IFNβ, is an essential defence.^343,344^ YAP negatively regulates IFN-β signalling. Mice with YAP deficiency showed increased IFN-β levels compared to control mice after being infected with vesicular stomatitis virus and herpes simplex virus type 1 (HSV-1), suggesting enhanced innate immunity. Importantly, YAP deficiency reduced the mortality of mice after HSV-1 challenge.^345^ In contrast, LATS2 could support innate immunity by increasing INF-β expression after human immunodeficiency virus-1 infection in vitro, and the loss of LATS2 impaired the innate immune response.^346^ Recently, LATS1 was shown to be essential in regulating the activity of type I IFN signalling.^347^

Furthermore, the Hippo pathway is important for the function of immune cells that take part in adaptive immunity. First, the Hippo component Mst1/2 could affect the proliferation of naïve T cells and the number of peripheral T cells. Although Mst1 deficiency did not change the process of T-cell development, it could decrease the thymic egress of T cells and increase the proliferation of naïve T cells. Double knockout of Mst1 and Mst2 reduced peripheral T cells, while the deletion of Mst2 alone did not significantly change peripheral T cell numbers.^348,349^ Besides, the Mst1 deficiency was shown to decrease the numbers of marginal zone B cells and memory B cells.^350,351^

In addition, among the immune cells that can be regulated by the Hippo pathway, Treg cells and Th17 cells are worthy of attention because of their close connection to autoimmune diseases.^352,353^ It was reported that Mst1-Mst2 was essential for maintaining the Treg pool, while TAZ contributed to the production of Th17 cells and the function of Tregs. Deletion of Mst1-Mst2 led to autoimmune diseases. However, TAZ knockout made the mice resistant to autoimmune encephalomyelitis.^354,355^ In the clinic, autoimmune manifestations were found in Mst1-deficient patients as well.^356^

### Therapeutic targeting of the Hippo pathway

The close connection between the Hippo pathway and various diseases indicates that the Hippo pathway is an appealing therapeutic target. Until now, no drug specifically targeting the Hippo pathway has been developed for clinical use, likely due to the relatively short history of this pathway. However, potential Hippo-targeted drugs have been widely investigated in both preclinical (Table 1) and clinical trials (Table 2).^357,358^ At present, the development of potential drugs mainly focuses on three aspects of the Hippo pathway, including Hippo core kinase activity/expression, downstream YAP/TAZ expression levels and YAP/TAZ-TEAD interactions.^7^ The Hippo core kinases inhibit YAP/TAZ to control its location and subsequently influence the expression of Hippo target genes.^114^ Thus, inhibitors of Hippo core kinases can be readily developed. However, inhibiting Hippo core kinases can result in YAP/TAZ activity, which is often associated with pathogenesis, particularly cancer. This could be a concern, although Hippo core kinase inhibitors may promote regeneration and wound healing. Furthermore, when YAP/TAZ translocates into the nucleus, the transcription and expression of Hippo-related genes depend on the interaction between YAP/TAZ and TEAD1-4.^148^ YAP-TEAD transcriptional activity can be suppressed by reducing the expression of YAP/TAZ, disrupting the YAP-TEAD interaction or inhibiting TEAD activity, which makes these potential targets interfere with the Hippo pathway.

**Table 1:** The potential drugs targeting Hippo pathway in preclinical trials

Mechanism		Drugs	Structure/sequence	Indications
YAP/TAZ nucleus/cytoplasm location	MSTs kinase activity inhibition	XMU-MP-1		Chronic and acute liver injury Protest cancer Breast cancer Autoimmune encephalomyelitis Cardiac hypertrophy
		SBP-3264		Acute myeloid leukaemia
	MSTs kinase expression inhibition	MST1/2-siRNA	MST1 (5’–3’ sense GAGUGUCAAUAUUGCGAGAtt) MST2 (5’–3’ sense CAAGAGUCAUGAAAAUUGUtt)	Deficiency of liver regeneration
	LATs kinase activity inhibition	TRULI		No certain indication so far
	Sav kinase expression inhibition	*Sav*-shRNA	Not post	Myocardial infarction Ischaemic heart failure
YAP-TEAD transcriptional activity regulation	YAP/TAZ expression inhibition	CA3		Oesophageal adenocarcinoma Osteosarcoma tumour
		YAP-siRNA	Not post	Glioblastoma Hepatocellular carcinoma Posterior segment neovascularization-related ocular diseases
		YAP-shRNA	Not post	Lung fibrosis
	YAP-TEAD interaction inhibition	verteporfin		Glioblastoma Breast cancer Hepatocellular carcinoma Renal interstitial fibrogenesis Glaucoma
		VGLL4-mimiking peptide	SVDDHFAKSLGDTWLQIGGSGNPK- TANVPQTVPMRLRKLPDSFFKPPE	Gastric cancer Colorectal cancer
	TEAD palmitoylation inhibition	Flufenamic acid derivative		Glioblastoma (in vitro)

**Table 2:** The drugs targeting Hippo pathway in clinical trials

Mechanism	Name (sponsor)	Phase	Indications	ClinicalTrials.gov Identifier
TEAD palmitoylation inhibition	VT3989 (Vivace Therapeutics)	Phase 1	Solid Tumour Mesothelioma	NCT04665206
	IK-930 (Ikena Oncology)	Phase 1	Solid Tumours Mesothelioma Epithelioid Hemangioendothelioma NF2 Deficiency YAP1 or TAZ Gene Fusions	NCT05228015
YAP antisense oligonucleotide	ION537 (Ionis Pharmaceuticals)	Phase 1	Advanced solid tumours	NCT04659096
Not been disclosed	IAG933 (Novartis)	Phase 1	Mesothelioma	NCT04857372

#### Hippo core kinase inhibition

Hippo core kinases can be manipulated by kinase inhibition or altering protein expression. MST kinase activity inhibitors are the most common Hippo core kinase activity inhibitors in preclinical studies. In particular, XMU-MP-1, an MST1/2 inhibitor identified by Fan et al.^201^ has been studied extensively in a variety of diseases. The initial effect of XMU-MP-1 was reported in liver repair and regeneration. Treatment with XMU-MP-1 can ameliorate both chronic and acute liver injury in mouse models in vivo.^201^ Combined with nanotechnology, Liu et al.^359^ loaded XMU-MP-1 in a novel nanohybrid to optimize efficacy. In vivo, the XMU-MP-1-loaded nanohybrid showed a longer inhibitory effect on p-YAP and better efficacy in acute liver failure than the free drug. In addition, in cancers such as prostate cancer and breast cancer, MST frequently plays a protumorigenic role. It was reported that XMU-MP-1 may have potential in treating prostate and breast cancer because XMU-MP-1 can inhibit proliferation in a variety of prostate and breast cancer cell lines.^360^ Moreover, in other mouse models, such as autoimmune encephalomyelitis and cardiac hypertrophy, XMU-MP-1 could relieve these conditions.^361,362^ However, XMU-MP-1 is not the most ideal inhibitor of MSTs because of its off-target activity.^201^ Many other MST inhibitors have been identified. Among them, SBP-3264, an MST1/2 small molecule inhibitor that was designed recently, could be used to treat acute myeloid leukaemia.^360,363^ In addition to MST inhibitors, Hippo core kinase inhibitors have not been extensively examined. Kastan et al.^364^ identified a potential inhibitor of LATS that was initially named TRULI. However, more details, including the crystallographic information of the binding site and the efficacy of the inhibitor in treating certain diseases, require further investigation.

Experiments to reduce Hippo core components rely on gene knockout/knockdown. The downregulation of multiple Hippo kinases has the potential to treat diseases. For example, it was reported that knocking down Sav significantly ameliorated heart failure.^365^ Consistently, studies in mice with ischaemic heart failure showed improved heart function after Sav knockdown.^301^ In partial hepatectomy mouse models, siRNA-mediated knockdown of MST1/2 efficiently induced liver regeneration.^330^

#### Inhibition of YAP/TAZ expression/activity

Modulation of YAP/TAZ protein levels or activity mainly involves two approaches in preclinical and clinical studies: pharmacological inhibition and genetic inhibition. CA3 is the representative pharmacological inhibitor of YAP expression that was identified through chemical library screening; however, the detailed mechanism has not been studied thoroughly.^366^ CA3 treatment reduces the growth of oesophageal adenocarcinoma and osteosarcoma.^366,367^

Genetic inhibition is another commonly used approach to decrease the expression of YAP/TAZ. siRNA therapy is an alternative to small-molecule inhibitors and has more specificity.^368^ The efficacy of YAP/TAZ-based siRNA therapy has been verified in a variety of animal models. D/R@Ang2-Lip+Au, a doxorubicin- and YAP-siRNA-loaded cationic liposome, was able to effectively inhibit glioblastoma.^369^ In addition, another YAP-siRNA-lipid nanoparticle repressed hepatocellular carcinoma in a mouse model.^370^ In addition to cancer treatment, YAP-siRNA was effective in treating posterior segment neovascularization-related ocular diseases.^371^ In addition to siRNA, shRNA is another common tool for genetic inhibition. Bleomycin-induced pulmonary fibrosis was attenuated when YAP was downregulated by AAV5-sh-YAP1 and AAV-miR-15a treatment.^310^ This proven efficacy and unique advantages make genetic inhibitors of YAP/TAZ an exciting potential development prospect. Most notably, thus far, ION537, an antisense medicine targeting YAP1, has been evaluated in phase I clinical trials. This is a great breakthrough for the development of a Hippo-targeted drug (NCT04659096).^372^

As awareness of the Hippo pathway continues to improve, new ways to inhibit YAP/TAZ have been discovered recently. SuperHippo, a designed WWC-derived protein, was shown to inhibit YAP/TAZ activity by inducing the phosphorylation of LATS1/2 due to the requirement of the WWC protein in the activation of LATS1/2.^31^

#### Inhibition of TEAD and/or the YAP-TEAD interaction

YAP-TEAD interaction inhibitors currently offer the diverse potential to target the Hippo pathway. Liu-Chittenden et al. found that verteporfin (VP) could bind to YAP and thereby disrupt its interaction with TEAD.^373^ In subsequent studies, VP was widely used to treat various diseases, including cancers,^374–378^ fibrotic diseases^379,380^ and glaucoma.^381^ Moreover, as a recognized YAP-TEAD interaction or YAP inhibitor, VP has been frequently used in studies of Hippo-related mechanisms.^382,383^ However, similar to other potential Hippo-targeted small molecule inhibitors, VP was shown to have off-target and YAP-independent toxic effects.^384^

Other widely discussed Hippo-targeted YAP-TEAD inhibitors are VGLL4 peptide mimetics. Based on the mechanism by which VGLL4 can bind to TEAD and compete with YAP/TAZ,^385,386^ VGLL4 peptide mimetics were designed to disrupt the YAP–TEAD interaction. Studies of their efficacy are mainly related to cancer treatment. For example, Super-TDU was designed by Jiao et al. to treat gastric cancer in mouse models. After Super-TD treatment, YAP target genes were suppressed, and the tumour was markedly remitted.^44^ In another study, Super-TDU was used to treat colorectal cancer. Similarly, tumour growth was significantly suppressed. Overall, VGLL4 peptide mimetics may be potential Hippo-targeted cancer drugs.^387^

With further exploration of the YAP–TEAD complex structure, the palmitoylation pocket of TEAD has become a new target for inhibiting the transcriptional activity of YAP–TEAD. Palmitoylation is a kind of protein modification that changes cysteine thiols of the substrate protein to thioesters with a palmitoyl group.^388^ In TEAD, it was previously found that the palmitate chain was inserted into a hydrophobic pocket of TEAD, and palmitoylation played an important role in the binding between TEAD and YAP/TAZ.^389^ The TEAD transcription factor is very unique in these properties and is not shared by any other transcription factors that normally are very difficult to target. In contrast, due to palmitoylation, TEAD is rather easy to target. Thus, there have been pharmaceutical efforts to develop TEAD inhibitors. Chloromethyl ketone 2, a derivative of FA that was identified in 2019, has been proven to disrupt TEAD4 binding to YAP1 by binding to the TEAD4 palmitate pocket. In vitro, the potential for treating glioblastoma has been demonstrated.^390^ Strikingly, three TEAD palmitoylation inhibitors have been examined in human clinical trials. VT3989 was developed by Vivice Therapeutics (NCT04665206), and the oral inhibitor IK-930 was by Ikena Oncology (NCT05228015).

## Conclusions and perspectives

Due to extensive basic research, the key components of the Hippo pathway network, including MST1/2, LATS1/2, MOB1A/B, SAV1, YAP/TAZ, and TEAD1–4, have been defined. Furthermore, other proteins, such as NF2, MAP4Ks, and WWC1/2/3, were revealed as important elements of the Hippo pathway. The main components and regulatory mechanisms of the Hippo pathway are already in place. The discovery of new components may further improve our understanding of the Hippo pathway in the context of many physiological and pathological processes. Additionally, although a number of upstream signals have been shown to influence the activity of the Hippo pathway, the comprehensive and meticulous interpretation of how these upstream cues are transmitted to Hippo components is incompletely understood.

Moreover, since the Hippo pathway is involved in the regulation of many crucial physiological processes, such as the proliferation and differentiation of cells, embryogenesis, and tissue regeneration, it is not surprising that dysregulation of the Hippo pathway is linked to an array of pathological consequences. Therefore, many therapeutic approaches that target Hippo core kinases, YAP/TAZ, or TEAD have been suggested for the treatment of various diseases.

Several strategies may improve the therapeutic value of current Hippo-targeted drug development efforts. First, combining Hippo-targeted therapies with advanced drug delivery systems, such as extracellular vesicles, nanoparticles, and polymeric vectors, could potentially increase the delivery efficiency of drugs. Second, a deeper understanding of the connection between the Hippo pathway and its upstream signals might provide novel perspectives on how the activity of the Hippo pathway can be manipulated. However, it is noteworthy that the Hippo pathway is not only implicated in the development of diseases but plays essential role in the maintenance of physiological homoeostasis. Thus, future drug development needs to examine ways to improve the efficacy of drugs while minimizing their adverse effects on the normal functions of the Hippo pathway.
