# Correction: The Hippo signalling pathway and its implications in human health and diseases

**DOI:** 10.1038/s41392-023-01682-3

**Published:** 2024-01-04

**Authors:** Minyang Fu, Yuan Hu, Tianxia Lan, Kun-Liang Guan, Ting Luo, Min Luo

**Affiliations:** 1 Breast Disease Center, State Key Laboratory of Biotherapy and Cancer Center, National Clinical Research Center for Geriatrics, West China Hospital, Sichuan University, No. 17, South of Renmin Road, 610041 Chengdu, China; 2 Department of Pediatric Nephrology Nursing, Key Laboratory of Birth Defects and Related Diseases of Women and Children, Ministry of Education, West China Second Hospital, Sichuan University, 610041 Chengdu, China; 3 Department of Pharmacology and Moores Cancer Center, University of California, San Diego, La Jolla, CA USA

**Keywords:** Molecular biology, Molecular medicine

Correction to: *Signal Transduction and Targeted Therapy* (2022) 7:376; 10.1038/s41392-022-01191-9 Published: 08 November 2022

After online publication of the article^1^, the authors noticed that inadvertent mistakes occurred in Figs. 2 and 3 that need to be corrected. In Fig. 2, one of the components of Hippo signalling pathway, TAZ, was miswritten as YAZ. The same miswriting was also occurred in ‘Mechanical cues’ section in main text (When myoblasts are in an elongated rectangular shape, the ratio of cytoplasmic to nuclear YAP/YAZ is increased). All the ‘YAZ’ should be corrected as ‘TAZ’. In Fig. 3, the ‘Annexin A_2_ in panel d should be corrected as ‘Annexin A2’, and HIF-α in panel e should be corrected as ‘HIF-1α’. The correct figure is provided as follows. The key conclusions of the article are not affected by these corrections. The original article has been corrected.

The authors apologize for any inconvenience caused to the journal and readers.
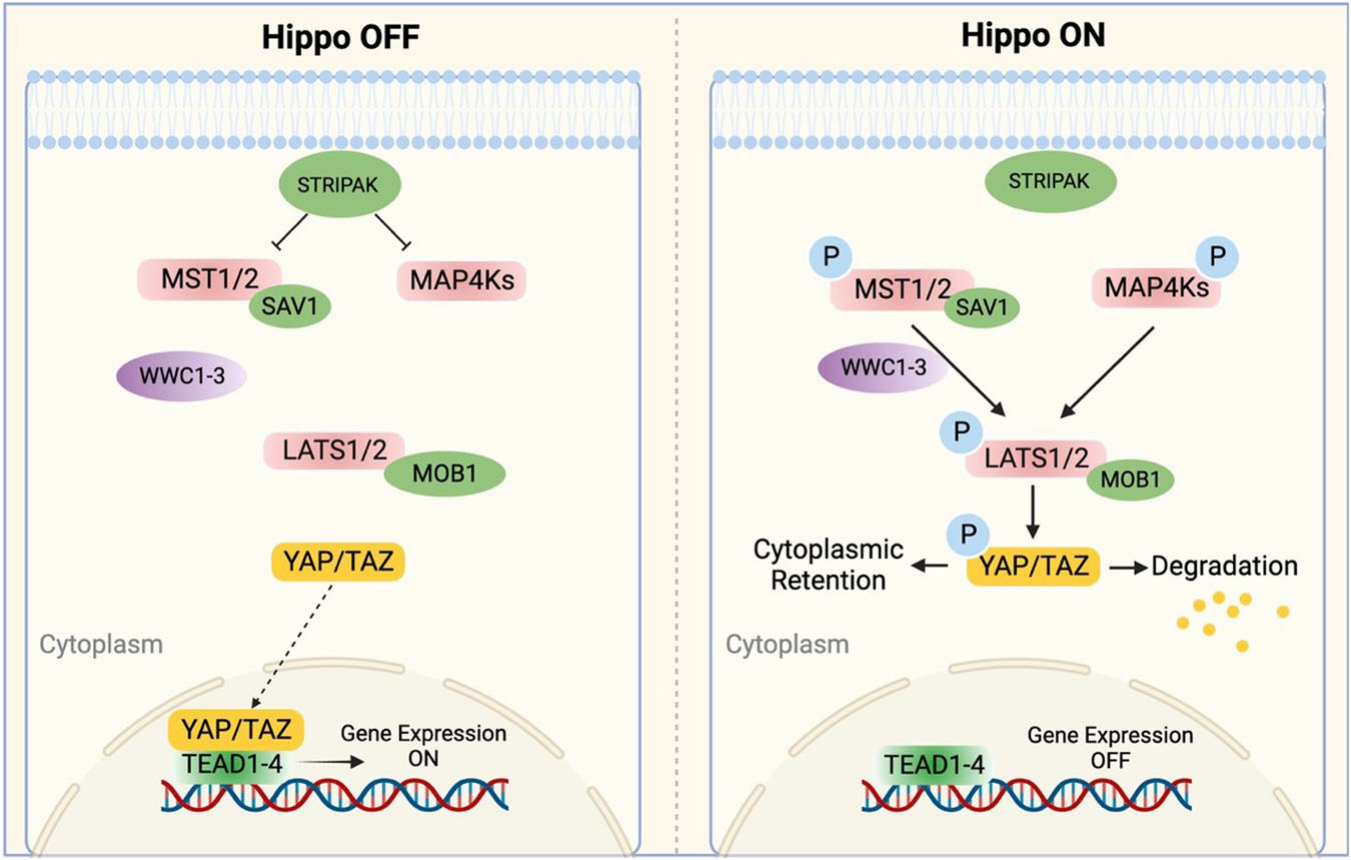


Fig. 2 The core Hippo pathway in mammals.

STRIPAK complex in the upstream regulates both MST1/2 and MAP4Ks. MAP4Ks or MST1/2 and its scaffold protein SAV1 could phosphorylate LATS1/2 and its scaffold MOB1 with the help of WWC1-3. The phosphorylated MOB1 can also directly promote the activation of LATS1/2 by inducing the conformational change of LATS1/2. The activated LATS1/2 phosphorylated and inactivated YAP/TAZ, preventing it from translocating into the nucleus and binding to transcription factors TEAD1-4
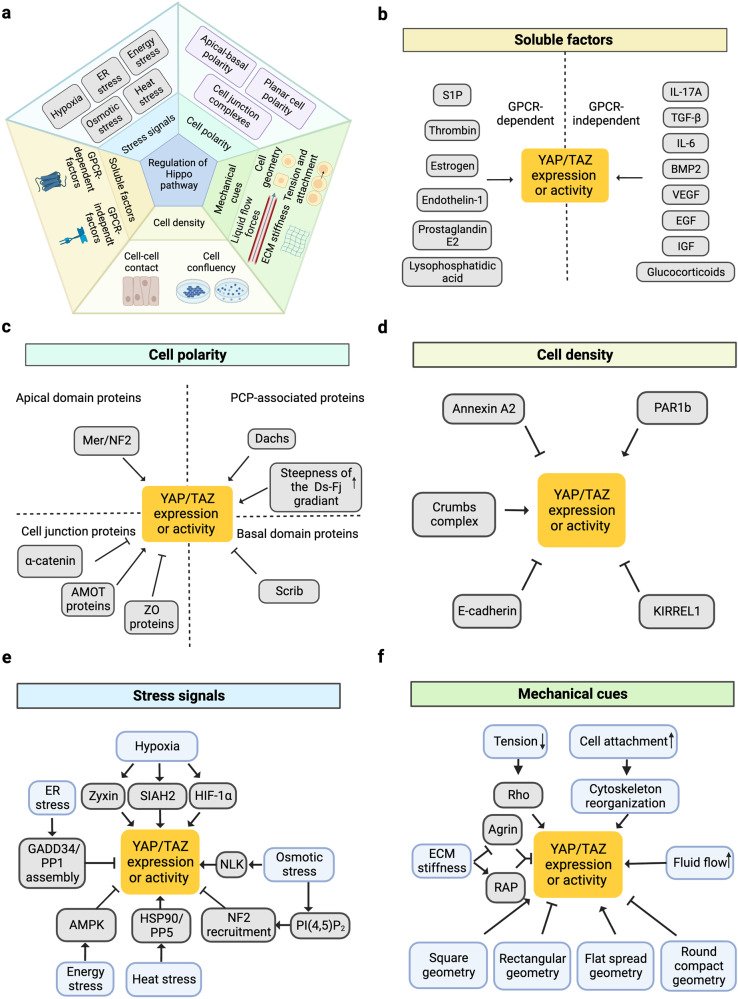


Fig. 3 Regulation of the Hippo pathway by upstream signals.

(a) Five subgroups of upstream signals including cell polarity, mechanical cues, cell density, soluble factors, and stress signals are responsible for the regulation of Hippo pathway. (b–f) The detailed upstream signals of Hippo pathway in every subgroup.
